# Peripheral and Central miRNA Signatures in Alzheimer’s Disease: Tissue-Specific Variability, Sex-Associated Differences, and Implications for Blood-Based Biomarkers

**DOI:** 10.3390/ijms27135990

**Published:** 2026-07-03

**Authors:** Amy S. Shiyab, Erin G. Reed

**Affiliations:** 1 School of Biomedical Sciences, Kent State University, Kent, OH 44140, USA; ashiyab@neomed.edu; 2 Department of Pharmaceutical Sciences, Northeast Ohio Medical University, Rootstown, OH 44242, USA

**Keywords:** Alzheimer’s disease (AD), microRNA (miRNA), extracellular vesicles (EVs), circulating biomarkers, amyloid-beta (Aβ), amyloid precursor protein (APP), neurofibrillary tangles (NFTs), four core genotypes (FCG), five familial AD mutations (5xFAD), X chromosome inactivation (XCI)

## Abstract

Alzheimer’s disease (AD) is a progressive neurodegenerative disorder characterized by cognitive decline and significant neuropathological changes. Early and accurate diagnosis remains a major challenge, highlighting the need for reliable, minimally invasive biomarkers. MicroRNAs (miRNAs), small non-coding RNAs that regulate gene expression, have emerged as promising candidates. Their expression is altered in the brains of AD patients, reflecting disease-specific pathological processes, and they are detectable in peripheral biofluids. However, discrepancies in miRNA profiles between the brain and the circulation, and between patient populations remain a significant limitation, raising questions about their origin, transport across the blood–brain barrier, and their reliability in reflecting central nervous system pathology. This review provides a comprehensive overview of current research comparing miRNA expression profiles in brain tissue and blood in AD, with a focus on their biological relevance, mechanisms of release and transport, and diagnostic potential. We also discuss the challenges associated with cross-tissue variability, methodological inconsistencies, and the need for standardized approaches. Finally, we highlight future directions, including multi-tissue analyses and integration with other noninvasive modalities, to improve the clinical utility of miRNA-based biomarkers in AD.

## 1. Introduction

### 1.1. Importance of Early AD Diagnosis

Alzheimer’s disease (AD) and its related dementias are projected to affect 153 million individuals worldwide by 2050, imposing an immense global health burden and significant emotional, social, and economic challenges on patients, families, and healthcare systems [1,2,3]. Despite decades of research, AD remains difficult to diagnose at its earliest stages. AD pathology can occur independently or with other neurodegenerative and vascular diseases, particularly in an aging brain. The pathologic processes of amyloid beta (Aβ) accumulation, tau hyperphosphorylation, synaptic dysfunction, and neuroinflammation begin decades before clinical symptoms emerge [4,5]. Therefore, differential diagnosis in a timely manner is critical for appropriate care, support, and individualized treatment plans [6,7,8]. However, current diagnostic methods rely heavily on invasive cognitive assessments and neuroimaging or cerebrospinal fluid (CSF) analyses, which can be expensive and inaccessible in many clinical settings. As a result, there remains an urgent need for noninvasive diagnostic tools capable of detecting AD before significant neuronal damage occurs.

Because clinical disease evolves over decades, the timing of diagnosis is important for accurate AD characterization. Early diagnosis improves accuracy, reducing risk of misdiagnosis or inappropriate treatment, especially for atypical or non-classical AD [7]. While typical AD is characterized by early memory impairment, with progressive decline in other cognitive domains, atypical AD often presents with visual/spatial problems, language difficulties, executive dysfunction, and behavioral symptoms [9]. These symptoms often overlap with other neurodegenerative dementias such as frontotemporal dementia, Lewy body dementia, and progressive aphasia [7,9,10].

It is therefore critical to identify and develop methods for early detection, shifting the basis of diagnosis from symptoms to biology, so that AD pathology, rather than its consequences, are detected [7]. Modeling studies have suggested that early diagnosis and intervention may delay a person’s time to institutionalization, resulting in societal cost savings, as well as slow both cognitive and functional decline. Additionally, newer disease-modifiable treatments require early diagnosis and intervention, providing added impetus to detect AD earlier [5,11,12,13]. For example, early-stage AD patients treated with Lecanemab or Donanemab showed significant cognitive and functional benefits with greater efficacy when treatment is initiated at earlier disease stages underscoring the importance of identifying AD at the mild or prodromal stage to maximize therapeutic benefit [14,15,16].

Beyond clinical outcomes, early diagnosis provides important psychosocial advantages. Major Alzheimer’s associations advocate for early diagnosis, allowing patients and families more time to plan, make informed decisions, and access support services, ultimately reducing emotional burden and improving quality of life [17,18,19,20]. Together, these findings highlight the importance of early detection for clinical management and downstream societal burdens.

### 1.2. Significance of Noninvasive Diagnostic Biomarkers

AD pathology begins years before clinical symptoms appear, with Aβ accumulation and tau dysregulation detected more than 15 years prior to diagnosis [21,22,23,24]. Currently, the only diagnostic tools available for AD are cerebrospinal fluid (CSF) biomarkers (e.g., Aβ, tau) and neuroimaging (e.g., PET scans), which are effective but often invasive, expensive, and not easily scalable [25,26]. Blood-based biomarkers, in contrast, are minimally invasive, comparatively inexpensive, and can be standardized allowing for early detection, monitoring, and broader clinical application [27,28,29,30].

Advances in highly sensitive assays now allow detection of molecular signatures of AD in peripheral tissues and other biofluids (e.g., blood, saliva), providing accessible alternatives for patients unable or unwilling to undergo lumbar puncture or PET imaging [30,31,32]. Beyond clinical utility, these biomarkers facilitate drug trials, mechanistic studies, and multi-modal research approaches by integrating with imaging, genetic, and cognitive data to enhance predictive accuracy [33,34].

### 1.3. MiRNAs as Potential Diagnostic Biomarkers in AD

Several aspects of microRNAs (miRNAs) contribute to their strong potential as diagnostic biomarkers. They have emerged as critical regulators of a wide array of biological processes including cellular metabolism and proliferation, apoptosis, neuronal gene expression and integrity, and brain morphogenesis [35,36,37,38,39,40,41]. Significantly, their dysregulation has been implicated in multiple aspects of AD pathology, including Aβ metabolism, tau phosphorylation, neuroinflammation, and synaptic dysfunction, as well as broader disruptions in cell signaling, transcriptional regulation, and structural pathways [42,43,44,45,46,47,48,49,50]. Shifts in their expression in neurodegenerative conditions like AD potentially alter cellular pathways, contributing to disease progression [51].

The literature suggests the existence of disease-specific miRNA signatures [31,39,41]. Some miRNAs such as miR-150-5p correlate with clinical and imaging markers of disease severity [45,52,53,54,55], whereas others like miR-483-3p demonstrate functional effects in experimental models, influencing neuronal survival, apoptosis, and AD-related protein expression [42,43,44]. The consistent dysregulation of these and other miRNAs across human and animal studies underscores their potential as biomarkers and therapeutic targets, though further validation is needed [39,40,41,56].

**Figure 1:**
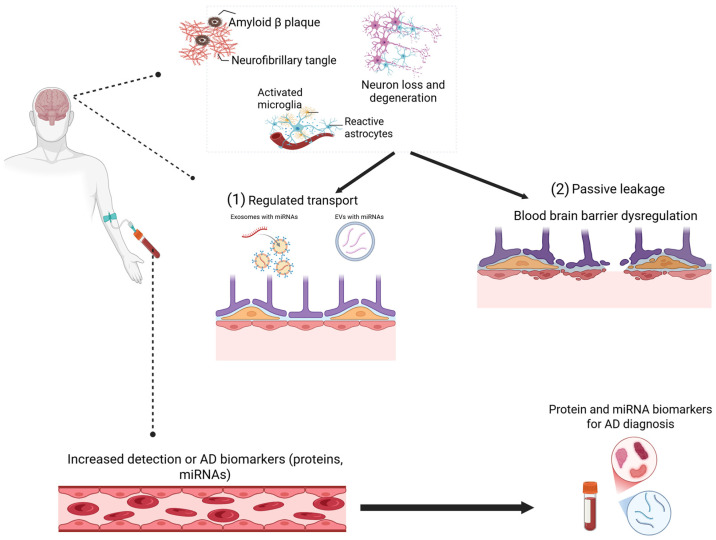
Proposed mechanisms linking brain miRNA dysregulation to peripheral biomarker detection in AD. AD-associated neuropathology, including Aβ plaque accumulation, NFTs, glial activation, and neuronal degeneration, alters miRNA expression within the CNS. These dysregulated miRNAs may enter the peripheral circulation through two non-mutually exclusive routes: (1) regulated export via EVs/exosomes and other carrier-associated transport mechanisms, and (2) passive leakage resulting from BBB dysfunction and increased permeability. Once in circulation, brain-derived miRNAs, together with other AD-associated biomolecules, may contribute to detectable peripheral biomarker signatures in blood. Created in BioRender. Shiyab, A. (2026) https://BioRender.com/mrpjwxe, accessed on 27 June 2026.

miRNAs are influenced by factors such as subcellular localization, relative abundance of both miRNAs and target transcripts, and the binding affinity between the two [57,58,59,60]. Moreover, miRNAs are remarkably stable due to their size and packaging in exosomes, vesicles, or protein complexes. They are resistant to RNase degradation, freeze thaw cycles and pH variations. Their stability and versatile roles in many biological processes make them ideal candidates as biomarkers for disease detection [61]. Additionally, their packaging and peripheral transport via exosomes and extracellular vesicles (EVs) may reflect brain-derived signals more faithfully than free circulating miRNAs because EVs can cross the blood–brain barrier and carry central nervous system (CNS)-derived cargo (Figure 1 [40,62]). Indeed, EV-miRNAs have demonstrated superior diagnostic performance compared to serum miRNAs, with some levels correlating with brain atrophy [40].

However, there are limitations to miRNAs as biomarkers, namely the context-dependent variability [63]. It is likely the variability observed in miRNA studies not only reflects isolated technical limitations, but also the context-dependent nature of miRNA expression itself. miRNA profiles are highly dynamic and shaped by multiple interacting biological variables, including tissue origin, disease state, sex, and experimental model, all of which influence their detectability and interpretation in biomarker studies.

### 1.4. Current Gaps, Purpose, and Scope of the Review

Despite the rapid expansion of miRNA research in neurodegenerative disease, significant knowledge gaps remain. There are inconsistent or conflicting miRNA signatures across tissues, cohorts, and experimental platforms, complicating standardization of biomarkers [45,64,65,66]. Peripheral miRNA expression does not always reflect molecular changes in the brain, where AD pathology originates [56,65,66,67,68,69,70,71]. Genetically engineered mouse models provide useful mechanistic insight but do not always fully recapitulate key features of human AD, limiting the translational validity of preclinical miRNA findings [72,73,74]. Additionally, sex, aging, and genetic variability influence miRNA expression and may contribute to inconsistent biomarker performance [75].

Standardization of sampling and analytical methods remains a major challenge. Variability in sample type, collection, processing, and quantification undermines reproducibility [75,76,77,78]. Many studies rely on small, often biased cohorts that are rarely reproduced in independent populations, with limited consideration of covariates such as sex, disease stage, or tissue type [75]. Brain miRNA studies are further complicated by under-sampling and evaluation of different brain regions [79,80].

Mechanistically, many miRNAs identified as potential candidates are derived from expression surveys with few providing possible mechanisms, such as how miRNAs affect Aβ processing, tau pathology, neuroinflammation or neuronal survival [76]. Without this data, biological relevance remains speculative. Additionally, despite growing appreciation for sex differences in AD manifestation and progression, very few studies looked at miRNA differences between men and women, and those that do are limited by unequal sex representation [75,76,81,82]. Taken together, the lack of reproducible validation and standardized protocols, limited large and balanced cohorts, and minimal mechanistic insight means that current miRNA signatures in AD must be interpreted with caution, rather than reliable diagnostic or prognostic tools. While miRNAs remain promising noninvasive biomarkers, existing research should be considered as preliminary until these gaps are addressed.

This review synthesizes current evidence on miRNA dysregulation across central and peripheral tissues in AD, with a particular focus on their potential as blood-based biomarkers (Figure 1). We compare miRNA expression patterns across different brain regions and peripheral biofluids, evaluate the consistency of these patterns between tissues, and consider possible mechanisms for how miRNAs might act across these different compartments. In addition, we examine miRNA alterations reported in commonly used AD mouse models and assess the influence of sex-specific differences on miRNA regulation. Finally, we highlight the key limitations and knowledge gaps in the field and propose future directions aimed at advancing the development of reliable and clinically useful miRNA-based diagnostic tools for AD.

## 2. MiRNAs and AD

MiRNAs are produced through a highly regulated biogenesis pathway involving DROSHA-, DGCR8-, and DICER-mediated processing before functioning as post-transcriptional regulators of gene expression [83,84,85]. Detailed information on miRNA biogenesis is available elsewhere [84,86,87]. These miRNAs act post-transcriptionally to regulate gene expression by binding target mRNAs and inhibiting translation or promoting mRNA decay [88,89,90].

### 2.1. Involvement of miRNAs in AD-Related Gene Expression

Beyond maintaining cellular homeostasis, miRNAs modulate the expression of important AD-related proteins like Amyloid Precursor Protein (APP), β-site APP-cleaving enzyme 1 (BACE1), and components of the γ-secretase complex (PSEN1/2), contributing to neuroinflammation [91,92,93,94,95].

Direct targeting of APP mRNA by miRNAs was first reported over a decade ago. Members of the miR-20a family (miR-20a, miR-17-5p, and miR-106b) regulate APP mRNA, with their expression patterns closely paralleling APP levels in the brain and in differentiating neurons, suggesting that changes in miRNA expression could influence APP levels during development and in disease [96]. Recent studies continue to show miRNA involvement in the APP amyloid pathway. For example, miR-298 reduces APP expression and Aβ production in human cell models, and miR-124 influences amyloidogenic pathways by altering APP-derived Aβ production in neuronal AD models [94,97,98]. Thus, through post-transcriptional regulation of APP expression, miRNA dysregulation could alter amyloid production and contribute to AD progression.

Multiple miRNAs exert context-dependent effects on BACE1 determined by their target and functional roles. Down-regulation of some miRNAs increases BACE1 expression to enhance Aβ production, while other miRNAs exhibit a more protective role, reducing Aβ accumulation [61,94,97,98]. MiRNAs regulate γ-secretase by targeting the 3′-UTR of *PSEN1*. For example, miR-3940-5p reduces *PSEN1* expression, lowering PSEN1 levels. Because PSEN1 is a critical component of the γ-secretase complex that cleaves APP to generate Aβ, this miR-3940-5p-mediated downregulation of PSEN1 decreases Aβ production [91,99]. MiR-29b-2-5p directly represses PSEN1 expression and its upregulation similarly reduces Aβ and improves cognition [100].

In addition to Aβ accumulation, miRNAs are implicated in the regulation of tau pathology at multiple levels, including expression, post-translational modification and protein clearance [101,102,103,104,105,106] demonstrating that miRNAs form an integrated regulatory network governing tau expression, phosphorylation state, and clearance, underscoring their central role in tau-mediated neurodegeneration in AD.

The inflammatory response to Aβ is mediated by glial cells, including microglia and astrocytes, exacerbating AD pathology [107,108,109,110]. With neuroinflammation representing a major component of AD pathology, understanding the regulatory mechanisms of miRNAs involved in inflammatory mechanisms would facilitate future disease interventions [111]. In fact, multiple miRNAs regulate immune signaling pathways, microglial activation, and cytokine expression. Many are upregulated in AD brain and CSF, and exert opposing immunomodulatory effects depending on the context [42,61,112,113,114], demonstrating miRNAs are not simply pro- or anti-inflammatory; rather that they act to fine tune immune responses, and when their regulation is disrupted in AD, can contribute to long-lasting, damaging neuroinflammation [92]. Overall, and because changes in these miRNAs can be detected in peripheral samples such as blood, studies often highlight their potential not only as regulators of amyloid pathology but also as accessible biomarkers reflecting disease-related molecular changes in the brain.

### 2.2. MiRNA Stability in Peripheral Fluids

Despite shared biological processes that regulate miRNAs in tissues and the circulation, miRNAs in the circulation are generally more stable compared to those in tissue cells for several reasons including their intrinsic decay rates, post-transcriptional modifications, protein interactions, packaging in EVs, and environmental conditions [115,116,117,118].

A relatively high intracellular concentration of miRNAs is often required for functional repression of multiple target transcripts, reflecting the broad regulatory potential of a single miRNA, so their stability is critical for achieving sufficient concentrations to engage multiple targets [119,120,121]. MiRNA half-life varies from minutes [122] to several weeks [119,122,123,124], and miRNAs rank among the fastest produced and longest-lived cellular transcripts, harboring hundreds to thousands of copies per cell at a steady state. Mature miRNAs are produced within minutes, revealing tight intracellular coupling of biogenesis. This biogenesis, however, can be selectively disrupted by alterations in microprocessor activity, DICER-dependent processing, RNA-binding proteins, and splicing-associated regulatory mechanisms, resulting in reduced maturation of specific miRNAs [84,125,126,127]. And because only one strand of the miRNA duplex is efficiently loaded into the miRISC complex due to selective strand loading, this can result in a significantly less active miRNAs in the cell [128,129].

Extracellular mature miRNAs in human blood plasma and serum are highly resistant to nucleases because they are associated with AGO proteins and are often packaged in EVs (referred to as exosome microvesicles), shielding them from degradation enzymes [115,130,131,132,133,134]. As a result, EV-derived and total circulating miRNA profiles may differ [135,136,137,138,139,140]. Although circulating miRNAs are generally considered stable due to protection by AGO complexes and extracellular vesicles, variability in reported stability under conditions such as freeze–thaw and temperature exposure likely reflects differences in pre-analytical handling, sample composition, and miRNA-specific susceptibility rather than true uniform degradation [117,131,141,142,143].

Overall, enhanced stability of peripheral miRNAs contributes to their slower turnover compared to miRNAs in actively proliferating cultured cells or progenitor stem cells and why they are more often used as biomarkers for disease detection [130,144]. These findings emphasize the remarkable protection that vesicles give to miRNA stability in the blood and even with that protection, how miRNA stability can vary.

### 2.3. Implication of Circulating miRNAs for AD Detection

Circulating miRNAs are altered in patients with AD or mild cognitive impairment (MCI), a condition where cognitive abilities are worse than expected for age but not severe enough to be classified as dementia and may be a step in developing AD or other dementias [39,145,146]. This suggests miRNAs could serve as minimally invasive blood-based biomarkers for early disease detection and progression monitoring [145,147,148,149,150,151].

There are distinct yet variable serum and plasma miRNA signatures in AD. miRNAs such as miR-181a, miR-181c, and miR-495 differentiate AD from MCI, while other circulating miRNAs are altered in both MCI and AD, with higher levels observed in individuals who progress from MCI to AD [39,41,45,48,71]. Importantly, overlapping miRNA alterations detected in both plasma and CSF further reflect underlying brain pathology [103,152,153]. Circulating miRNAs exhibit good diagnostic performance in AD [147,154]. There are also disease specific miRNA expression patterns in plasma exosomes, with a panel of miRNAs predicting AD with an Area Under the ROC Curve (AUC) of 0.85–0.88 [66,155]. Among these, dysregulation of miRNAs involved in immune signaling pathways and microglial activation correlates with neuroinflammatory processes and AD biomarkers such as t-tau, Aβ-40, and Aβ-42, reinforcing their potential for early diagnosis [39,66,156,157,158,159,160].

Overall, evidence supports circulating miRNAs as a compelling class of blood-based biomarkers for AD detection. Their stability in peripheral biofluids, association with AD-related biological pathways, and moderate diagnostic accuracy, particularly when used in multi-miRNA panels, all underscore their potential clinical value. Despite significant challenges in their applicability, including methodological heterogeneity and limited disease specificity, they are still valuable targets with remarkable potential as biomarkers for AD detection and diagnosis.

## 3. Sex Differences and miRNA Regulation in AD

### 3.1. Role of Sex in AD

AD is sexually dimorphic in terms of disease risk and prevalence, manifestation, and pathology. We refer the reader to several recent reviews detailing these differences [161,162,163,164,165,166,167,168]. In short, women make up a disproportionate number of cases due in part to a longer lifespan and living longer following a diagnosis. They tend to exhibit cognitive reserve despite greater amyloid and tau pathology, inflammation, and a greater impact of risk factors such as APOE ε4 [169,170,171]. Importantly, sex-stratified analyses often reveal effects that are missed when men and women are analyzed together [169,170,172,173,174,175,176].

Menopause correlates with biomarker changes and brain atrophy [177,178,179]. Estrogen is neuroprotective and plays crucial roles in the brain. It enhances synaptic plasticity (especially in the AD-relevant hippocampus and prefrontal cortex), promotes mitochondrial efficiency and antioxidant defenses, regulates APP processing, reduces Aβ accumulation, and modulates tau phosphorylation, potentially slowing NFTs. When estrogen levels drop at menopause, neurons lose this protection, and the brain regions vulnerable to AD (hippocampus, temporal lobes) begin to show accelerated atrophy, with increased CSF tau, p-tau, and sometimes amyloid burden [180,181,182].

While much attention has focused on the influence of menopause and declining estrogen levels in women, hormonal changes in men may also contribute to AD risk. Testosterone, one of the major sex hormones in men, is essential for the maintenance of secondary sexual characteristics and fertility, but studies have also found that it exerts a neuroprotective effect by improving synaptic signaling and counteracting neuronal death [183]. Unlike the quick hormonal shift observed in women, men tend to experience a more gradual decline in testosterone, which has been associated with cognitive decline, and neurodegeneration [183,184,185,186].

### 3.2. Sex Hormones and miRNA Expression in AD

Estrogens and androgens exert widespread regulatory effects on gene expression in the brain, including modulating miRNA networks, making them highly relevant to AD [75,82,184]. Therefore, it is important to understand how these hormones shape miRNA expression and contribute to interpreting sex-specific disease mechanisms and diagnostic signatures.

Estrogen is produced throughout the body [187,188,189], and decreases significantly as people age [190,191,192]. Estrogen acts through three main receptors, the alpha, beta, and G protein coupled estrogen receptors, known to modulate miRNA profiles directly (i.e., miRNA transcription) and can influence microRNA biogenesis through regulation of components of the Drosha and Dicer processing machinery [193,194,195]. Estrogen depletion, including ovariectomy (OVX), leads to widespread alterations in brain miRNA expression, with negative downstream effects on pathways involved in cognition, stress responses, synaptic plasticity, and neuroprotection [196,197,198,199,200,201]. Specific estrogen-sensitive miRNAs such as miR-218 have been implicated in neuronal function and AD related tauopathies [202]. Others like miR-106a were shown to be neuroprotective through STAT3 suppression, which is involved in inflammation and neurodegenerative signaling [190,203]. Previously, miR-106a was observed to regulate APP expression in the brain and influences neuronal differentiation [204]. Together, these findings support a model where hormonal status shapes miRNA regulatory networks in the CNS, providing a molecular framework through which sex hormones may contribute to sex-specific vulnerability and progression in AD.

Androgens and their signaling have also been implicated in AD. Androgens may exert a protective role against AD by reducing Aβ production and accumulation, and lower levels of testosterone may be associated with an increased risk of AD [186,205,206,207]. Furthermore, androgen signaling may modulate tau pathology by indirectly regulating pathways involved in tau phosphorylation, such as intracellular kinase activity and cell signaling networks [172,208,209]. Interestingly, early experimental studies demonstrated that androgen signaling may contribute to diabetes induced AD by influencing metabolic regulation, insulin signaling, and neuroinflammatory pathways, suggesting that reduced androgen levels may exacerbate diabetes-associated brain changes, thereby increasing susceptibility to neurodegeneration and cognitive decline, although these findings have not been recently validated [210,211,212,213,214].

Overall, these findings suggest that sex hormones can and do contribute to AD risk in a bidirectional and sex-specific manner, supporting the premise that disruption of both estrogenic and androgenic signaling can shift brain homeostasis toward a more neurodegeneration-prone state.

### 3.3. X-Linked miRNAs and AD

The X chromosome harbors roughly 120 miRNAs, accounting for nearly 10% of all known human miRNAs, versus only a few on the Y chromosome, and represents a disproportionately high number for the X chromosome compared with most autosomes [215,216,217]. Of the X-linked miRNAs identified, about half are grouped in clusters and may have evolved faster compared to autosomal miRNAs, suggesting they may have specialized functions in sex-specific regulatory programs, including development, immunity, and brain function, though many X-linked miRNAs remain poorly characterized [218,219,220].

**Figure 2:**
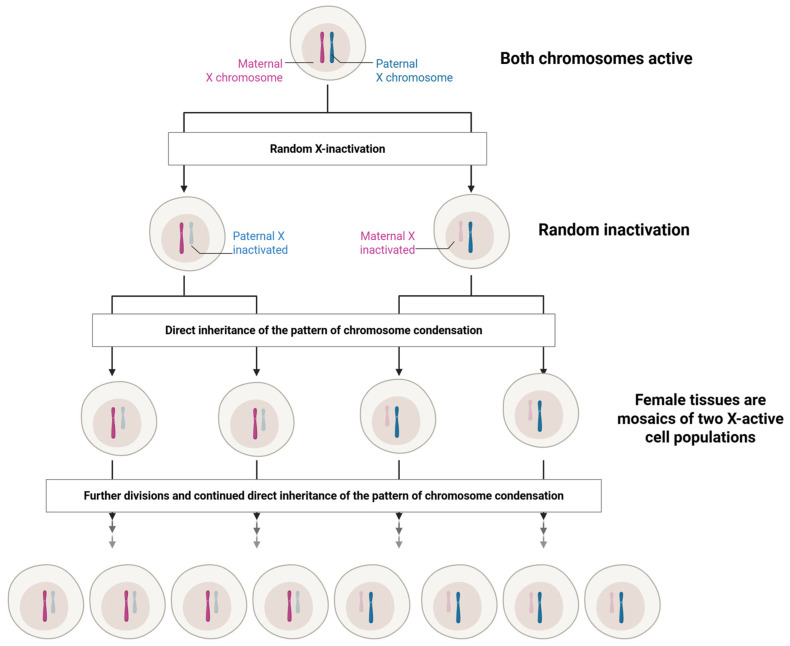
Random X-Chromosome Inactivation and Clonal Maintenance During Development. The figure shows X-chromosome inactivation is initiated randomly in early embryonic cells, leading to mosaic gene expression that is stably maintained through subsequent cell divisions. Created with BioRender. Shiyab, A. (2026) https://BioRender.com/zhc66p8, accessed 27 June 2026.

X-chromosome inactivation (XCI) is a fundamental epigenetic process that ensures dosage compensation between sexes by transcriptionally silencing one X chromosome in XX-bearing cells [221,222,223,224]. However, this inactivation is not always complete or consistent, where large proportions of X-linked genes escape inactivation, and inactivation patterns may become skewed or destabilized with aging, known as skewed XCI [224,225,226,227,228]. Normally, XCI is random, where roughly 50% of cells inactivate the maternal X and 50% inactivate the paternal X [227]. When one X chromosome is preferentially inactivated in a much larger proportion of cells, it gives a pattern different from the ideal 50:50 ratio [227,229,230], with different proportions of the female population typically showing skewed ratios of 70:30, 80:20 and 90:10 [229] (Figure 2). Because specific genes that reside on the X chromosome and their genetic and epigenetic regulation may play a role in AD, XCI escape and skewing may contribute to the sex bias and disease processes observed in AD [176].

X-linked microRNAs have been implicated in AD through multiple expression profiling studies showing sex-dependent differences in miRNA signatures in both brain and peripheral tissues. Several X-chromosome linked miRNAs, such as miR-221/222, miR-98, and miR-532-3p, are dysregulated in AD and are associated with key disease-relevant pathways such as neuroinflammation, synaptic dysfunction, BBB integrity and neuronal stress responses [231,232,233,234,235,236,237,238,239]. These findings suggest that X-linked regulatory miRNAs may contribute to molecular heterogeneity in AD and potentially to the observed sex differences in disease susceptibility and progression. Although the functional roles of many X-linked miRNAs in AD remain incompletely defined, emerging transcriptomic and small-RNA sequencing data support their involvement in AD-related neurobiological pathways.

To address the relevance of X-linked miRNAs to AD, studies using mouse models like the Four Core Genotypes (FCG) model are particularly informative, as they allow separation of chromosomal sex effects from gonadal sex. This framework is critical for interpreting sex differences in AD-relevant molecular pathways, including miRNA regulation, as it distinguishes whether observed differences are driven by XX vs XY complement or by hormonal environment. Within this context, X-linked miRNAs can be more directly evaluated as contributors to sex-biased neurodegenerative processes rather than as purely correlative biomarkers.

## 4. AD Mouse Models and Translational Relevance for Biomarker Development

Mouse models are essential for studying AD pathophysiology and preclinical therapies, because they recapitulate key disease features such as Aβ accumulation, tau pathology, synaptic dysfunction, and neuroinflammation. Common models include the 5xFAD, APP/PS1, 3xTg-AD, and PS19, while sex-focused models such as the FCG, OVX, and 4-vinylcyclohexene diepoxide (VCD) allow the investigation of sex chromosome and ovarian hormone effects. Because no single model fully captures human AD, each have distinct strengths and limitations that must be considered in data interpretation. Table 1 summarizes major AD and sex-based mouse models and their translational relevance. Combining AD models with sex-focused and/or FCG models enables deconstructing the roles of gonadal hormone and sex chromosomes on miRNAs and disease processes. Collectively, these models support the identification of sex-dependent miRNA signatures and assessment of their translational relevance to human brain and peripheral biomarkers in AD.

## 5. MiRNA Expression Patterns in the Human Body

MiRNA expression patterns in the human body are tissue specific, and in the brain, region specific, reflecting differences in cellular composition, neuronal vulnerability, and pathological burden [79,324,325,326]. MiRNA dysregulation is inconsistent in AD, varying across brain regions and tissues, contributing to differential pathology [79,113,324,327,328,329].

There is, however, a degree of overlap and directionality of change in miRNA expression in peripheral blood, CSF and brain tissues from AD patients that varies across studies, reflecting both biological complexity and methodological heterogeneity [46,55,113,330,331]. The translation of peripheral miRNA profiles to CNS pathology is complicated by tissue- or disease state-specific variability, despite some miRNAs showing consistent changes. Additionally, major methodological differences further complicate direct comparisons of miRNA profiles. Finally, despite well-established sex differences in AD susceptibility, progression, neuroinflammation, and hormonal regulation, relatively few studies explore miRNA expression in a sex-specific manner [68,75,113,332]. Collectively, these findings support the need for integrated and sex-specific approaches to studying miRNA expression across the brain and periphery in AD. Selected examples in Table 2 below highlight the extent of this variability.

## 6. Potential Mechanisms Linking Brain and Peripheral Tissue

MiRNAs produced in the CNS can be detected in both the brain and peripheral fluids (blood, plasma, and serum), suggesting mechanistic crosstalk between the brain and periphery. In AD, several X-linked miRNAs exhibit dysregulated expression in both compartments [55,62,64]. And although direct evidence for sex-dependent miRNA trafficking between the CNS and periphery remains limited, some studies found sex-dependent differences in extracellular vesicle biology, such as increased brain extracellular vesicle secretion and altered vesicle trafficking dynamics in females relative to males, as well as increased tau-related biomarkers [172,387,388,389]. Similarly, in women undergoing the menopausal transition, neuron-enriched extracellular vesicles isolated from plasma displayed hormone-sensitive alterations in miRNA cargo associated with synaptic, inflammatory, and neurodegenerative pathways, further supporting the possibility that sex hormones modulate CNS-periphery extracellular miRNA signaling [389].

### 6.1. Transport Across Blood–Brain Barrier

Transport of miRNAs between the CNS and the periphery provide a mechanistic link between brain pathology and circulating biomarkers [390,391]. The blood–brain barrier (BBB) is a tightly regulated interface between the brain parenchyma and circulation [392,393] that becomes compromised in AD, facilitating altered molecular exchange [394]. MiRNAs can travel between these compartments through several mechanisms [99,395], with the strongest evidence for EVs, RNA-binding proteins, and lipoproteins, and weaker support for passive diffusion and tunneling nanotubes [396,397].

EVs are lipid bilayer–bound particles released by all cell types that mediate intercellular communication by transporting proteins, lipids, mRNAs, and miRNAs [386,398,399]. They are broadly classified into exosomes, microvesicles, and apoptotic bodies based on their biogenesis. Exosomes, the most extensively studied subtype, originate from endosomal trafficking via multivesicular bodies that fuse with the plasma membrane to release vesicles into the extracellular space [237,400,401].

As discussed above, EVs protect miRNAs from degradation and enable their stable transport in biofluids, facilitating CNS–periphery communication. In the nervous system, EV-mediated miRNA transfer contributes to the regulation of the BBB integrity, inflammation, and neuronal function, making them particularly relevant in AD [390,391,398,402,403]. In AD, there are some overlapping miRNA signatures between brain tissue, CSF and EVs, however most studies do not distinguish vesicle-associated from non-vesicle-associated miRNA populations, limiting mechanistic interpretation [62,375,400,404,405,406]. Additionally, neuronal-derived EV miRNAs in serum reflect early AD-associated brain changes, supporting their potential utility as biomarkers [70,99,407,408]. Collectively, these findings suggest that circulating miRNA profiles may partially reflect underlying brain pathology in AD; however, the extent of this “mirroring” is likely modulated by BBB integrity, selective extracellular vesicle loading, and peripheral contributions, which together shape the final circulating miRNA expression profile.

### 6.2. Inflammation and Immune Response on Circulating miRNAs

Recent reviews on miRNA regulation of innate immune signaling highlight the differential expression of miRNAs in the peripheral circulation that may respond to AD progression, and therefore serve as potential diagnostic biomarkers [31,55,113,409]. However, miRNAs do not simply reflect AD processes, but can actively modulate immune responses by engaging receptors such as TLRs [342,344,410,411]. Studies in AD show microglial-derived EVs contain specific miRNAs capable of activating TLR8, a key RNA sensor in innate immunity, prompting a proinflammatory response [114,412].

Additionally, several circulating inflammatory miRNAs directly influence signaling pathways downstream of TLRs, with implications for the systemic pro-inflammatory state associated with an increased risk of AD. For example, miR-146a targets key adapters like IRAK1 and TRAF6 in the TLR/NF-κB signaling cascade and modulates microglial polarization and inflammatory signaling [159,343,344,351,413,414]. Several studies exploring miRNAs modulating this process, known as “inflamma-miRNAs”, found plasma levels of several miRNAs were significantly elevated in AD patients compared with healthy matched controls [152,335,415]. And because many of these miRNAs regulate inflammatory signaling pathways implicated in AD pathology, their detection in the peripheral circulation may reflect ongoing neuroinflammatory activity within the brain. These findings suggest that circulating miRNAs may not only be passive biomarkers but may actively influence immune and inflammatory pathways with changes that may be detectable in the blood.

Together, these observations indicate that circulating miRNAs may arise from multiple sources, including CNS export, peripheral tissue expression, BBB-related transport processes, but also may contribute to or be a result of inflammation in the brain This heterogeneity complicates the direct interpretation of peripheral miRNA profiles as specific proxies for brain-derived molecular changes in AD.

## 7. Diagnostic Potential of miRNAs in AD Detection

As previously discussed, current diagnostic methods rely largely on clinical assessment and a limited set of biomarkers, and existing treatments provide only symptom relief without treating disease root cause or its progression. Having established that miRNAs reflect disease-specific changes in AD, their stability and accessibility in peripheral biofluids, coupled with consistent dysregulated patterns observed in blood, CSF, and brain tissues in numerous studies, it positions them as promising noninvasive diagnostic tools. Key miRNAs, including miR-132, miR-146a, miR-34a, and miR-125b, and miR-206 among others highlighted in this review, show reproducible alterations that may mimic changes observed in the brain. These reasons make them compelling candidates not only for early detection but also for potential therapeutic intervention [92,147,416].

Throughout this review, we have demonstrated that miRNAs are key functional players in AD, prompting the development of miRNA-based therapeutics aimed at correcting dysregulated gene networks [43,416,417]. There are two main strategies: (1) miRNA mimics or agonists which aim to restore downregulated protective miRNAs by mimicking the naturally occurring miRNA, and (2) antagomirs or antagonists, which inhibit or block pathogenic miRNAs contributing to disease progression [159,418,419].

Preclinical studies have demonstrated promising results. For example, miR-124, miR-188-5p, and/or miR-23b-3p mimics enhance synaptic plasticity, reduce Aβ accumulation, and improve cognitive function in AD models [420,421,422,423]. Alternately, antagonists targeting overexpressed miRNAs such as miR-206 or miR-142-3p alleviate cognitive deficits and dampen neuroinflammatory signaling [419,424,425,426]. Importantly, many of these miRNAs target multiple AD-relevant pathways simultaneously, offering the potential for multi-faceted disease modulation.

Despite this potential, several critical challenges must be addressed to enable clinical translation. Efficient delivery remains a primary obstacle, as miRNA mimics and antagomirs must cross the BBB and achieve targeted uptake in specific neural cell populations while minimizing off-target effects. Although chemical modifications and encapsulation approaches can enhance stability and delivery efficiency, the susceptibility of synthetic oligonucleotides to degradation in circulation remains a significant concern [427,428,429,430].

Specificity presents an additional limitation, given that a single miRNA can regulate hundreds of transcripts, increasing the likelihood of unintended downstream effects [431,432,433,434,435]. Furthermore, the potential for immunogenic responses, particularly with repeated or long-term administration, warrants careful evaluation [436,437,438]. To address these barriers, a range of delivery platforms are being evaluated, including lipid nanoparticles, viral vectors, and exosome-mediated systems, each offering distinct advantages in terms of stability, targeting precision, and safety profiles. Although these strategies have yet to achieve routine clinical implementation, they underscore the potential of miRNA-based therapeutic regulation as a novel, mechanism-driven approach for AD management [430,439,440,441].

In addition to clinical limitations, technical challenges remain substantial. First and foremost is the lack of methodological standardization across studies. Substantial variability exists in detection platforms, qRT-PCR, microarray technologies, and next-generation sequencing, as well as in the selection of biological matrices (e.g., plasma, serum, CFS, and exosome-derived samples). Additional inconsistencies arise from differences in RNA isolation protocols, normalization strategies, and pre-analytical variables such as sample collection procedures, storage conditions, and susceptibility to hemolysis. Collectively, these significantly influence measured miRNA expression profiles, contributing to inter-study discrepancies, reducing reproducibility, and ultimately limiting the reliability of miRNA-based diagnostics in clinical settings [40,45,66,113,147,416,442,443].

Although miRNAs show good overall diagnostic potential, their reported performance varies widely. Meta-analyses indicate pooled sensitivity and specificity values around 0.80–0.88, but individual studies often report conflicting results for the same miRNA [147,154]. Furthermore, many AD associated miRNAs are not disease specific, instead participating in common biological pathways such as neuroinflammation, oxidative stress, and apoptosis. Thus, similar miRNA expression patterns are observed in other neurodegenerative and systemic diseases and this overlap reduces their ability to reliably distinguish AD from conditions such as Parkinson’s disease or other dementias [147,444,445].

Although limited and not directly validated, there are implications that miRNA-based biomarker development in AD may be sex dependent, as several dysregulated miRNAs differed between males and females indicating the underlying molecular mechanisms in AD may vary by sex. These differences may influence biomarker reliability, diagnostic accuracy, and therapeutic targeting as well. Therefore, consideration of sex as a biological variable will be important for the development and interpretation of miRNA-based biomarkers in AD [45,75].

## 8. Future Directions

Despite significant advances in understanding miRNA dysregulation in AD and utility as biomarkers, several critical gaps remain. Current studies often focus on single tissues or biofluids, and methodological variability complicates the comparison of results across laboratories [150,446]. Moreover, while circulating miRNAs show promise as noninvasive detectors, their relationship to CNS pathology is not fully established [40,70]. Future research should focus on combining data across these tissues to find miRNAs in the blood that truly reflect what is occurring pathologically in the brain [64,393,447].

Although several recent studies have identified overlapping miRNA signatures between central and peripheral tissues and demonstrated associations with established AD biomarkers, larger longitudinal studies are needed to validate these findings and determine their clinical utility [55,64,152,156,448,449]. Combining miRNA profiling with complementary approaches, including transcriptomics, proteomics, neuroimaging, and other fluid biomarkers, may further improve diagnostic accuracy and provide a more comprehensive understanding of disease progression. Ultimately, translating these findings into clinical practice will require standardized analytical protocols, multicenter validation studies, and well-designed clinical trials to establish the diagnostic and therapeutic utility of miRNA-based biomarkers [450,451].

### 8.1. Standardize Testing Methods

Measuring miRNAs and quantifying them consistently remains a critical challenge, limiting their reproducibility and clinical translation. Studies have highlighted that pre-analytical variables significantly influence circulating miRNA profiles, contributing to variability across studies [118,446,452,453]. Furthermore, different RNA extraction protocols and analytical platforms (e.g., RT-qPCR, microarray, next-generation sequencing) result in inconsistent detection and quantification of miRNAs [454,455,456,457]. Another major unresolved issue is the lack of universally accepted normalization strategies, as commonly used reference controls can vary across experimental conditions, leading to differing results even within the same dataset [332,446,458,459,460]. Additionally, the absence of standardized reproducible protocols for circulating miRNA quantification further complicates cross-study comparisons and validation [446,461,462]. Collectively, these findings underscore the need for consistency in all the above-mentioned approaches to enable reliable comparison across studies and facilitate the clinical implementation of miRNA-based biomarkers.

### 8.2. Incorporate Other Noninvasive Modalities

Integration of noninvasive approaches such as saliva biomarkers, advanced imaging and proteomics should also be explored as a complement to improving the accuracy and clinical relevance of biomarker-based assessments [463,464,465,466,467]. Saliva contains miRNAs and other molecules related to AD, like Aβ or tau. While total tau is decreased, p-tau and Aβ42 are significantly increased in the saliva of AD patients [468,469,470], making them promising candidates as noninvasive indicators reflecting central pathology and warrant systematic validation in large cohorts and longitudinal designs [468,471,472].

Furthermore, advanced neuroimaging modalities, including MRI and PET, remain essential for linking peripheral miRNA profiles to structural and molecular brain changes. Multimodal approaches integrating imaging with blood-based biomarkers show improved predictive performance compared to single modalities, with associations between circulating miRNAs and amyloid, tau, and neurodegeneration imaging markers [152,473,474,475,476]. These findings highlight the value of combining imaging and peripheral biomarkers to improve early detection and refine disease characterization.

Proteomic profiling further complements miRNA-based approaches by capturing dynamic changes in protein expression associated with AD pathology. Alterations in blood and CSF proteomes have been linked to disease progression and neuronal injury, and integrating proteomic data with miRNA and imaging biomarkers enhances sensitivity and specificity for early detection and longitudinal monitoring [477,478,479,480,481,482,483,484].

This review would not be comprehensive without emphasizing the critical importance of considering sex as a biological variable as these challenges are addressed and future approaches are developed. As the field advances toward the standardization and validation of miRNA biomarkers, as well as their translation into clinical trials and therapeutic applications, sex must remain an integral component at every stage of investigation. Incorporating sex-specific analyses will be essential for improving the accuracy, reproducibility, and clinical relevance of findings, while also supporting the development of more precise and individualized diagnostic and therapeutic strategies. Ultimately, acknowledging and systematically evaluating sex-associated differences will strengthen the reliability of miRNA-based approaches and enhance their potential to advance personalized medicine in Alzheimer’s disease.

## 9. Conclusions

Circulating miRNAs show great promise as noninvasive biomarkers for AD with the potential to improve early detection, monitor disease progression, and guide personalized interventions. miRNAs participate in key pathogenic processes, including amyloid processing, tau pathology, inflammation, and sex-specific regulatory mechanisms, and their expression patterns differ between the CNS and peripheral tissues, often overlapping with disease-relevant brain changes. Studies in AD mouse models further support the translational relevance of miRNA research and provide mechanistic insight into how miRNAs are transported and regulated across tissues, including EVs and immune-mediated pathways.

Despite these advances, significant challenges remain. Sex differences add an additional layer of complexity, highlighting the need to consider hormonal and genetic factors when interpreting miRNA profiles. Furthermore, ethical considerations must be considered with early diagnostic strategies, particularly regarding the psychological impact of predictive biomarker testing. Together, these strategies provide a comprehensive roadmap for translating miRNA research into practical clinical applications, supporting personalized medicine approaches, and potentially improving outcomes for individuals at risk for or living with AD.

## Figures and Tables

**Table 1:** Overview of Alzheimer’s disease and sex-based experimental mouse models. Relevant references in parenthesis.

Model	Genetic Features	Main Pathology/ Timeline	Key Strengths	Limitations	Sex Differences	Translational Relevance
5xFAD	Overexpresses human APP and PSEN1 mutations associated with familial AD (APP: K670N/M671L, I716V, V717I; PSEN1: M146L, L286V) on congenic C57BL/6J background [240]	Rapid amyloid pathology beginning ~1.5 months; strong Aβ42 deposition, gliosis, synaptic degeneration, cognitive decline, and notable neuronal loss [240,241,242]	Robust and early amyloid pathology; strong neuroinflammation; useful for studying Aβ deposition, oxidative stress, innate immunity, behavioral deficits, and therapeutic interventions [243,244,245,246,247,248,249]	Does not develop robust NFTs or extensive cerebrovascular pathology; limited amyloid–tau interaction; phenotype influenced by transgene dosage and inheritance patterns [242,250,251,252,253]	Females show greater APP expression, increased Aβ burden, stronger inflammation, and detectable behavioral deficits [254,255,256,257,258]	Strong biomarker relevance with PET-detectable amyloid, cerebral hypometabolism, and overlap with human AD proteomic signatures; useful for serum biomarker discovery [259,260,261,262]
APP/PS1	Human APP Swedish mutation plus mutant PSEN1 [263,264]	Plaques develop in hippocampus and cortex around ~6 months; synaptic dysfunction and impaired LTP occur early [265,266,267,268,269]	Reliable amyloid deposition; strong glial activation; good for studying amyloid processing, neuroinflammation, synaptic dysfunction, and therapeutic testing [267,270,271]	No robust tau pathology or NFTs; overexpression-based familial AD model does not fully mimic sporadic late-onset AD [272,273]	Females often show greater cortical plaque burden and regional neuronal changes [271,274]	Valuable for amyloid PET tracer development, CSF/blood Aβ studies, inflammatory biomarkers, and preclinical therapeutic testing [263,275,276]
PS19	Overexpresses human 1N4R tau with P301S mutation under murine PrnP promoter [277,278]	Progressive tau hyperphosphorylation, tau aggregation, filamentous inclusions, gliosis, and neurodegeneration in hippocampus, cortex, brainstem, and spinal cord [277,279,280]	Strong tauopathy model; useful for studying tau aggregation, propagation, synaptic dysfunction, neurodegeneration, and microglial responses [281,282,283]	No amyloid pathology; based on frontotemporal dementia mutation rather than AD; pathology varies with strain, sex, and environment [72,279,284]	Sex and background strain can influence severity and timing of pathology [285,286,287]	Useful for studying tau-driven neurodegeneration and p-tau biomarkers, though limited by non-physiological tau overexpression [279,288,289]
3xTg-AD	Carries mutant APP Swedish (KM670/671NL), PSEN1 M146V, and tau P301L [290,291]	Intracellular Aβ accumulation begins at 2–6 months; extracellular plaques at 6–12 months; tau pathology at 12–20 months [292,293]	Develops both amyloid and tau pathology; useful for studying amyloid–tau interactions, cognitive decline, and synaptic dysfunction [290,294]	Less neuronal loss than human AD; familial mutation model does not fully reflect sporadic AD [290,291]	Age- and sex-specific inflammatory and pathological trajectories reported [295,296]	Valuable for studying combined amyloid/tau pathology and testing therapies targeting both hallmarks [297,298,299]
Four Core Genotype (FCG)	Sry deleted from Y chromosome and inserted onto autosome, producing XX and XY mice with either ovaries or testes [300,301]	Separates effects of sex chromosomes from gonadal hormones [300,301]	Powerful tool for studying sex chromosome vs hormonal influences on brain, immunity, and AD-related biology [301,302]	Genetically complex; requires larger sample sizes and complex statistics; does not independently model amyloid or tau pathology [301,303,304,305]	Sex chromosome complement and gonadal hormones independently and interactively shape brain and immune function [301,302,305,306]	Useful for mechanistic studies of sex-dependent immune and brain differences relevant to AD [302,305,306,307]
AD + FCG Crosses (5xFAD/FCG, APP/PS1/FCG)	Combination of AD transgenic models with FCG system [302,308]	Allows investigation of independent and interactive effects of sex chromosomes and hormones on AD pathology [302,304,308]	Enables controlled study of sex-dependent amyloid burden, microglial activity, neuritic dystrophy, and immune signaling [302,304]	Literature remains limited; technically and statistically complex [302,304]	Demonstrates independent effects of sex chromosome complement and gonadal hormones on AD pathology [302,304]	Enhances translational relevance by incorporating biological sex mechanisms into AD pathology and biomarker studies [302,304]
OVX (Ovariectomy) Model	Surgical removal of ovaries causing abrupt ovarian hormone depletion [309,310,311]	Produces anxiety-like behavior, depressive-like behavior, memory impairment, altered estrogen receptor expression, increased insoluble Aβ, and impaired microglial responses [309,310,311]	Useful for studying effects of ovarian hormone loss on cognition, neuroinflammation, and AD pathology [309,310,311]	Abrupt hormone loss does not fully mimic natural menopause [309,310,312,313]	Models hormone depletion rather than intrinsic chromosomal effects [309,310,311]	Widely used to study menopause-related contributions to AD risk and hormone replacement effects [309,314,315]
VCD Menopause Model	Chemical induction of gradual ovarian follicle depletion using 4-vinylcyclohexene diepoxide [316,317]	Gradual hormone loss resembling natural menopause; increased Aβ accumulation and region-specific glial activation [316,317]	Better mimics natural menopausal transition compared to OVX [318,319]	Chemical induction may alter systemic physiology beyond ovarian effects [320,321]	Focuses on gradual hormonal decline and associated brain changes [316,317,322]	Useful for studying menopause-associated neurobiological and AD-related processes [322,323]

**Table 2:** Comparison of Central and Peripheral miRNA Expression. Relevant references in parenthesis.

miRNA	Primary Function in AD	Key Pathways/ Mechanisms	Findings in Different Samples	Relevance in AD	Sex Differences in AD
miR-146a	Brain-specific inflammatory miRNA associated with neuroinflammation [333,334]	Regulates neuroinflammation, synaptic function, mitochondrial health, neuronal survival, and Aβ/NFT-related pathways through inflammatory feedback signaling [156,159,334]	Dysregulated expression is stage- and tissue-dependent, with consistent alteration in CSF, variable changes in plasma/serum (often increasing early and decreasing in later stages), and differential hippocampal and cortical expression [156,327,335,336,337,338,339]	Candidate biomarker and mechanistic regulator of inflammatory pathways in AD based on CSF detectability and inflammatory modulation [340]	Emerging evidence suggests that miRNA-146a dysregulation and inflammatory signaling pathways differ between males and females with AD [75,335,341]
miR-155	Pro-inflammatory miRNA involved in neuroinflammation and innate immune signaling in the CNS [342,343,344]	Targets SOCS1, SHIP1, and c-Maf, enhancing IL-1β, IL-6, and TNF-α signaling; promotes microglial activation, synaptic dysfunction, and impaired Aβ clearance [343,344,345,346,347]	Dysregulated in serum of AD patients, upregulated in hippocampus in inflammatory models, deletion reduces insoluble Aβ and alters synaptic pruning [343,344,347,348,349,350]	Supports the role of inflammatory miRNA networks in AD pathogenesis and biomarker development [348,351,352,353]	Evidence suggests that miR-155 expression exhibits sex-associated differences in immune cells, and its central role in neuroinflammatory signaling [350,354,355]
miR-29a/b	Amyloid-regulatory miRNA family generally considered protective in AD [100,356,357,358]	Regulates BACE1 expression and amyloidogenic APP processing [357,359,360,361,362,363]; also implicated in neuronal maturation, dendritic spine morphology, apoptosis, and synaptic function [364]	Downregulated in AD brain and peripheral blood [359,360,365]; some studies reported increased CSF levels with technical limitations noted [337]	Demonstrates context-dependent miRNA regulation affecting amyloid deposition, neuroinflammation, and cognition, supporting relevance as both mechanistic regulator and biomarker	Sex-stratified transcriptomic studies demonstrate that AD-associated miRNA expression profiles differ between males and females [75,366]
miR-132	Neuroprotective neuronal miRNA involved in synaptic plasticity and neuronal maintenance [367,368,369]	Associated with amyloid tau expression, phosphorylation, aggregation, autophagy, cognition, and neuronal survival [370,371,372,373]	Consistently downregulated in AD brains [53,79,329,367,372,374]; reduced circulating levels associated with cognitive decline and AD biomarkers [374,375]; miR-132 mimics improved cognition and tau metabolism in AD mouse models [372,373,376,377]	Strong mechanistic and biomarker candidate due to consistent links with tau pathology and neurodegeneration [373,376,377]	No direct sex-specific findings or sex stratified analyses found
miR-221	X-linked miRNA involved in inflammatory signaling, vascular dysfunction, and neuronal repair [324,378]	Associated with tau, amyloid, BBB integrity, pericyte survival, neuronal health, inflammation, and repair pathways [156,324,379]	Downregulated in blood and serum of AD patients [231,379]; overexpression restored BBB integrity and reduced neuronal loss in animal models [379,380,381]; inconsistent findings in AD brain tissue [79]	Relevant peripheral biomarker candidate due to detectable dysregulation in circulation [156,324,378]	X-linked localization may contribute to sex-specific regulatory differences observed in AD
miR-98	X-linked miRNA implicated in amyloid regulation, inflammatory signaling, and neuronal stress [234,237,238]	Regulates APP processing, Aβ production, tau phosphorylation, oxidative stress, mitochondrial dysfunction, apoptosis, and NF-κB-associated inflammatory pathways [234,237,238,382,383]	Large-scale AD findings remain inconsistent; studied primarily in peripheral biofluids as a potential biomarker [383,384,385,386]; Decreased in hippocampal AD samples [382]	Associated with amyloid metabolism, neuronal stress responses, and disrupted synaptic signaling in AD [237,238,382]	X-chromosomal localization supports investigation into sex-biased AD vulnerability and sex-specific disease mechanisms

## Data Availability

No new data were created or analyzed in this study. Data sharing is not applicable to this article.

## Abbreviations

The following abbreviations are used in this manuscript:

AD	Alzheimer’s Disease
miRNA	microRNA
EV	Extracellular vesicle
BBB	Blood–brain barrier
CSF	Cerebrospinal fluid
APP	Amyloid precursor protein
Ago2	Argonaut 2
DGC8	DiGeorge syndrome critical region gene 8
PSEN	Presenilin
TREM2	Triggering receptor expressed on myeloid cells 2
NFTs	Neurofibrillary tangles
TLR	Toll like receptor
FCG	Four Core genotype
5xFAD	Five familial AD mutations
CNS	Central nervous system
XCI	Chromosome inactivation
P-tau	Phosphorylated tau
PET	Positron Emission Tomography
SOCS1	Suppressor of Cytokine Signaling
IRAK1	Interleukin-1 Receptor-Associated Kinase 1
IGF-1	Insulin-like Growth Factor 1
TLR8	Toll-like Receptor 8
